# Mechanism of remodeling and local effects in vivo of a new injectable cosmetic filler

**DOI:** 10.1038/s41598-023-36510-9

**Published:** 2023-06-13

**Authors:** Likui Sun, Xiaoxia Sun, Wenting Ruan, Guoxi Che, Fuyu Zhu, Chenghu Liu, Min Wan

**Affiliations:** Shandong Institute of Medical Device and Pharmaceutical Packaging Inspection (National Medical Products Administration Jinan Quality Supervision and Inspection Center for Medical Devices), which is NMPA Key laboratory for Safety Evaluation of Biomaterials and Medical Devices, Key lab of biological evaluation of medical devices of Shandong province, Jinan, 250101 China

**Keywords:** Biomaterials, Tissue engineering, Biomedical engineering

## Abstract

By studying the local effects of a new type of injectable cosmetic filler implanted into the animal to explore the mechanism of remodeling and cosmetic effect of this kind of product. Take 12 rabbits and select 4 implantation points on both sides of the spine, respectively, and implant the test sample (PLLA) and negative control sample (HDPE) into the subcutaneous tissues on both sides. In the same way, take another 12 rabbits and implant the marketing control sample (cross-linked sodium hyaluronate) and negative control sample (HDPE) into the subcutaneous tissues on both sides. The animals were executed at 1 week, 4 weeks, 13 weeks and 52 weeks respectively, and Hematoxylin–Eosin staining, Masson trichrome staining and immunofluorescence staining were performed to characterize the local effects in vivo and the expression of type I collagen (Col. I), type III collagen (Col.III) and matrix metalloproteinase 9 (MMP-9). Good histocompatibility of the test sample and the marketing control sample were found. The foreign body reaction of marketing control sample was more intense than that of the test sample after 13 weeks. The foreign body reaction of testing sample was more intense after 52 weeks, while that of the marketing control sample was more stable. With the process of tissue repair, the collagen fibers of test samples and marketing control samples gradually increased after implantation. Type I collagen was mainly found inside the fiber capsule, while type III collagen was mainly found outside. The positive expression of matrix metalloproteinase 9 gradually increased, the positive expression of test samples increased significantly after 52 weeks, while that of marketing control samples did not change significantly. Good histocompatibility of PLLA filler is found. Matrix metalloproteinase 9 participates in foreign body reaction and collagen formation, which can reflect the process of tissue remodeling.

## Introduction

Injection cosmesis refers to injecting injectable material directly into a local or specific part of the human body, which is to modify the appearance or shape of the human body by the injection method. A standard injectable product in the market today is the hyaluronic acid product, a unique macromolecular linear polyanionicmucopolysaccharide widespread in various organisms and tissues. It is readily degraded in living organisms, which needs to be improved and derivatized to reduce its degradation rate. However, the novel injectable cosmetic filler poly (L-lactic acid) (PLLA) is derived from α- A family of biodegradable synthetic polymers with excellent biocompatibility, hydroxy acids have been widely used for dissolvable sutures, bone nails, and facial implants. Fibroblast activity can be stimulated by polyglactin to cause replacement growth of collagen and other connective tissue fibers. In 1999, new-fill, a new filler made from PLLA, was licensed and placed on the market in Europe, followed by PLLA (trade name: Sculptra) in 1999, also being certified in Europe for the treatment of symptoms such as wrinkling, scarring, skin aging. In 2004, the FDA approved Sculptra for treating AIDS-related lipoatrophy, with additional approval in 2009 for correcting mild to moderate nasolabial folds and other facial wrinkling indications in healthy applicants. The published research shows PLLA injection causes subclinical foreign body inflammation at the injection site, and the particles are encased and stimulate fibrous tissue proliferation and type I collagen deposition in the extracellular matrix^1^.Meanwhile, Bohnert et. reported that after one or more sessions of treatment, the accumulation of hyperplastic collagen fibrillar protein gives the natural filling effect that patients want to see^2^.The improving effect of PLLA on facial volume loss and wrinkles makes it gradually an injectable product with a significant post-hyaluronic acid effect (Table 1).

**Table 1:** Type I collagen expression at the injection site of filler in each group at different time points (${\overline{\text{x}}} \pm {\text{s}}$).

Groups	1 week	4 weeks	13 weeks	52 weeks
Test sample	20.69 ± 1.72	27.26 ± 1.30	31.73 ± 2.55	55.62 ± 1.88^a^
Control	44.13 ± 3.45	54.11 ± 3.48	64.86 ± 1.57	81.22 ± 2.41^b^

^**a**^Represents a significant difference between the sample group at 52 weeks and 13 weeks (p < 0.05) .

^**b**^Represents a significant difference between the control group at 52 weeks and 13 weeks (p < 0.05) .

After implantation of biomaterials, the implant degrades in vivo at different rates, products, and debris, potentially leading to inflammatory cell aggregation, foreign body giant cell formation, macrophage activation, and fibrosis^3,4^. Adhesive macrophages and multinucleated foreign body giant cells actively secrete matrix metalloproteinases to regulate wound healing, foreign body response, angiogenesis, and fibrous encapsulation around biomaterial implants^5^, and MMPs can degrade the structural components of the extracellular matrix and cell surface, among which MMP9 belongs to the family of gelatinases, a variety of cytokines and their receptors, collagen, gelatin Fibronectin and others can all serve as their substrates, which can upregulate expression in the early stages of inflammation and promote the integration and repair of host tissues with degradable materials^6^. This study addresses the current state of the art in evaluating the safety and efficacy of novel injectable cosmetic fillers in China by observing the tissue response at various times after in vivo injection of poly-L-lactic acid and the changes in type I collagen (col. I ), type III collagen (col. III) and matrix metalloproteinase 9 (MMP-9) expression from the histological and immunological levels to provide insight into the mechanism of action of such products, which elaborate the mechanism of this type of products in tissue remodeling and injection cosmesis, providing a reference for the development and regulation the products of injection cosmesis (Table 2).

**Table2:** Type III collagen expression at the injection site of filler in each group at different time points (${\overline{\text{x}}} \pm {\text{s}}$).

Groups	1 week	4 weeks	13 weeks	52 weeks
Test sample	17.54 ± 0.96	22.22 ± 0.93	35.87 ± 2.08	45.88 ± 0.47^c^
Control	44.48 ± 2.52	56.88 ± 1.86	65.31 ± 2.21	72.66 ± 1.39

^c^Represents a significant difference between the sample group at 52 weeks and 13 weeks (p < 0.05) .

## Result

### Observations of general indicator

After the operation, the mental status of the animal, drinking diet, and fecal traits were typical and healthy.

### Pathological examination of gross

**Figure 1:**
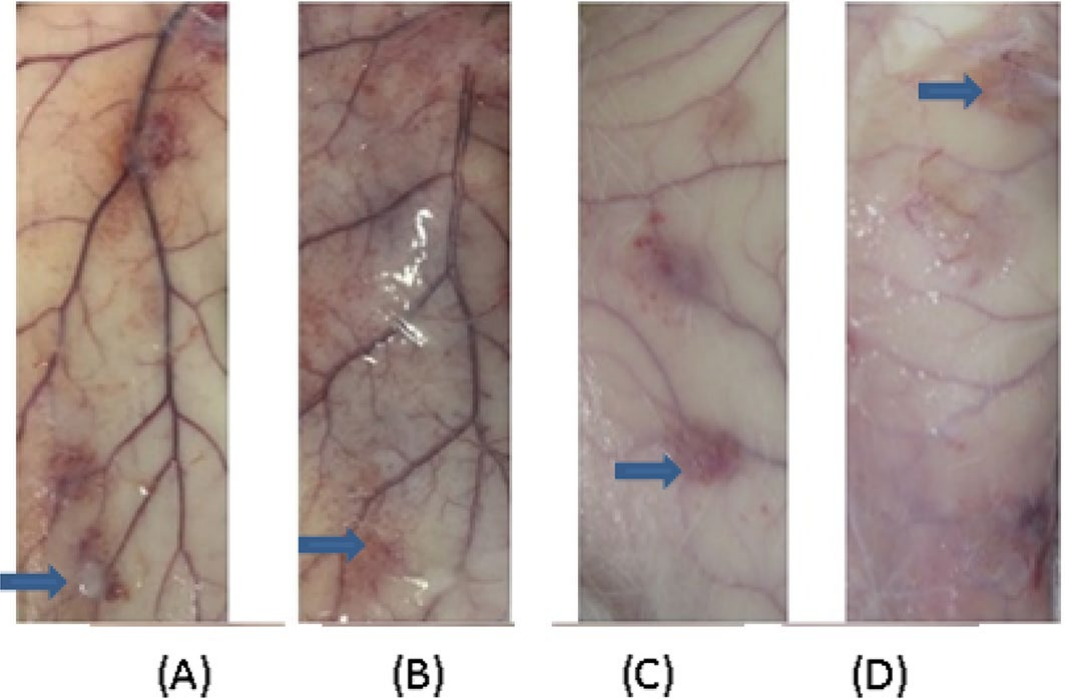
Gross observation of injection sites of test samples at different time points after the operation. (: the samples). The subcutaneous implantation for 1 week, 4-week, macroscopic observation shows that the samples have a pale yellow gel shape, the volume is not significantly changed compared with before, 13-week, 52-week visible samples and subcutaneous tissue are closely connected, the volume is not changed much compared with before. (**A**) 1 week, (**B**) 4-week, (**C**) 13-week, (**D**) 52-week.

**Figure 2:**
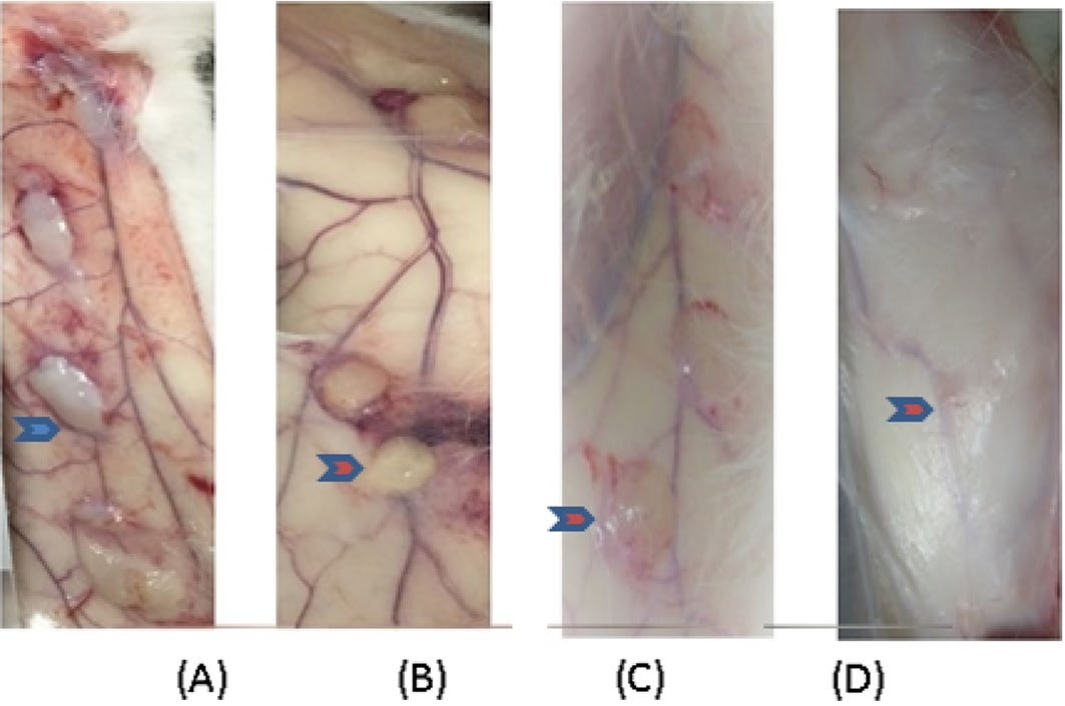
After subcutaneous implantation for one week, macroscopic observation, the visible samples were milky gelatinous, and the volume had no noticeable change. 4 weeks, 13 weeks, macroscopic observation, the visible samples were pale yellow gelatinous; the volume had little change than before; 52 weeks macroscopic observation, the visible samples were white membranous spread in the subcutaneous tissue, with no apparent protrusion of the surface. Gross observation of injection sites of marketed control samples at different time points after surgery. (: the control samples).

The subcutaneous implantation sites of test samples and marketed control samples were observed macroscopically at 1, 4, 13, and 52 weeks after surgery, showing no structural abnormalities in the animals (See Fig. 1, Fig. 2).

### Histopathological examination

#### Subcutaneous implantation for 1 week

**Figure 3:**
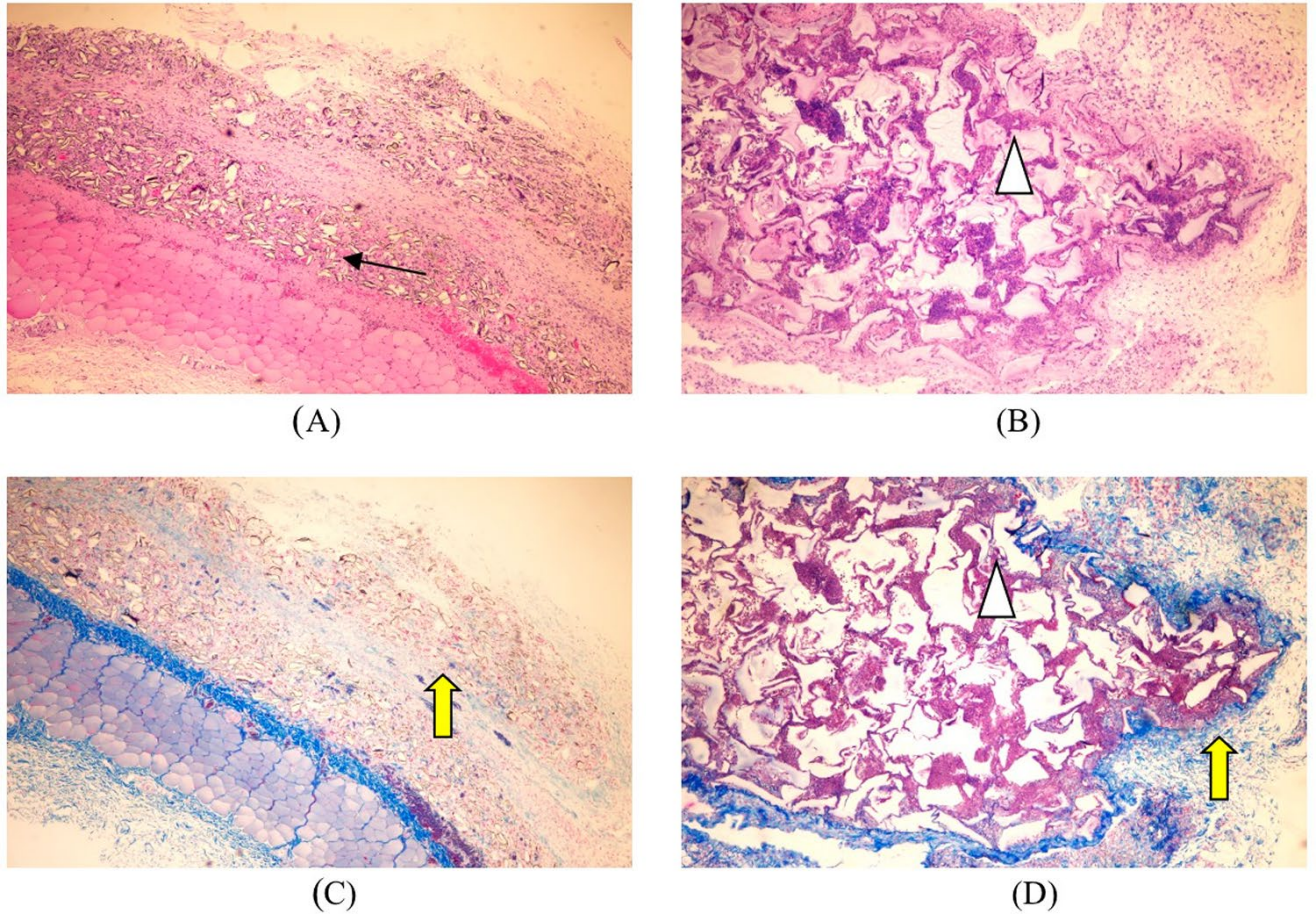
The result of Subcutaneous implantation for 1 week. (↑: test sample, : control sample, : collagen fibrils). (**A**) The test sample set 1W (he, × 40), (**B**) listed control group 1W (he, × 40), (**C**) test sample set 1W (Masson, × 40), (**D**) listed control 1W (Masson, × 40).

Hematoxylin eosin staining: the test sample group microscopic observation shows a large number of test sample particles distributed in the loose connective tissue of the subcutis (↑ identification in Fig. 3), surrounded by some inflammatory cells, macrophages, and lymphocytes infiltration, no significant change in volume compared to before. Microscopic observation of the marketed control sample group showed many gels distributed in the loose connective tissue beneath the skin (identified in Fig. 3), surrounded by many inflammatory cells, macrophages, and lymphocyte infiltration, and no significant change in volume compared to before (Table 3).

**Table 3:** Matrix metalloproteinase-9 expression at the injection site of filler in each group at different time points (${\overline{\text{x}}} \pm {\text{s}}$).

Groups	1 week	4 weeks	13 weeks	52 weeks
Test sample	19.79 ± 1.83	22.57 ± 2.99	23.99 ± 1.37	37.29 ± 1.59^d^
Control	54.43 ± 4.22	61.21 ± 0.78	67.50 ± 2.85	56.16 ± 3.46^e^

^**d**^Represents a significant difference between the sample group at 52 weeks and 13 weeks (p < 0.05) .

^**e**^Represents a significant difference between the control group at 52 weeks and 13 weeks (p < 0.05) .

Masson trichrome staining: collagen fibrils appeared blue (identified by yellow arrows in Fig. 3). In the test sample group, a small number of collagen fibers can be seen scattered in distribution, hydrophobically uneven, and arranged differently; The collagen fibers in the marketed control group 1 week after surgery were mainly distributed around the implantation point, and a small amount was distributed among the gels.

**Figure 4:**
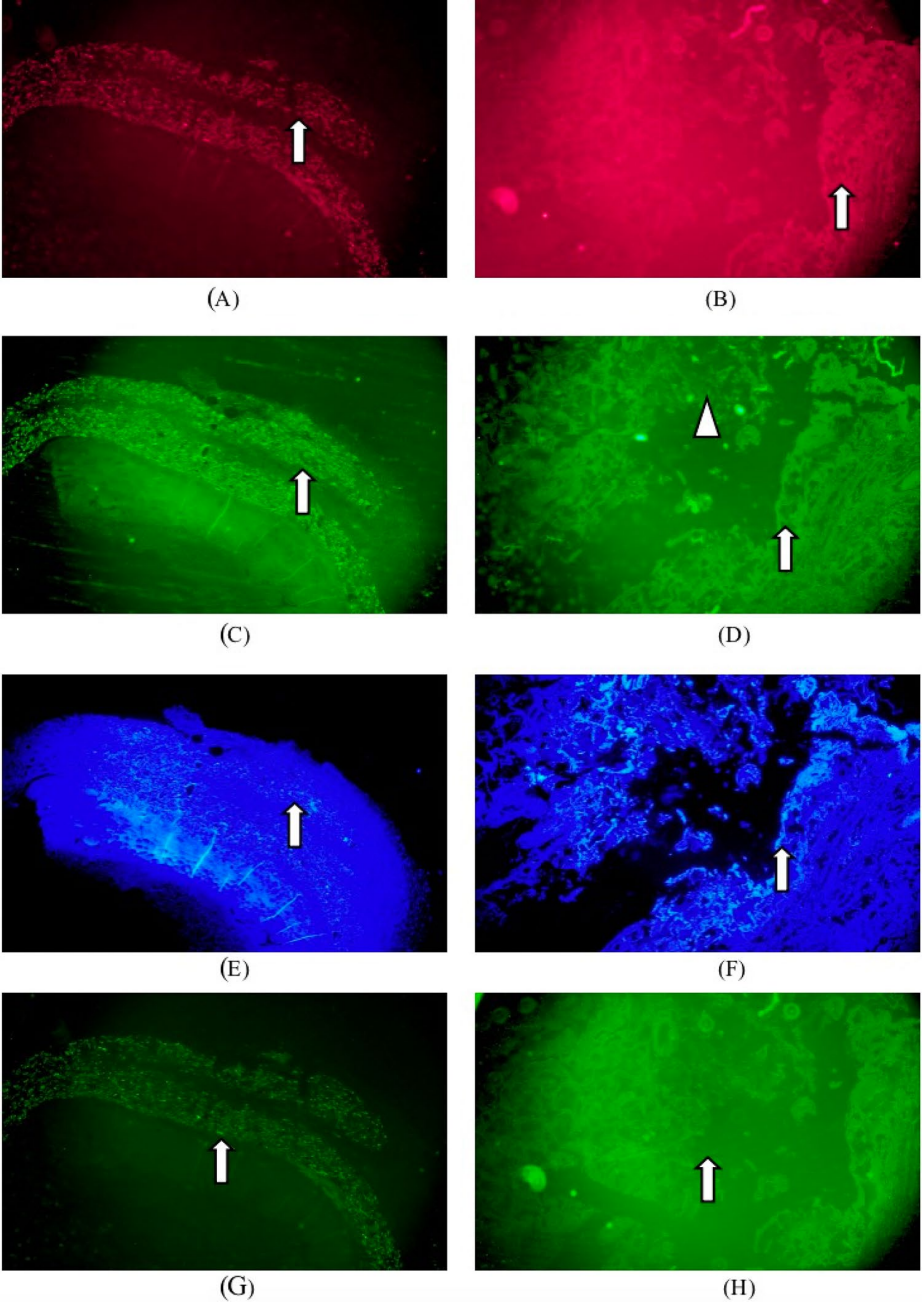
Immunofluorescence staining one week after implantation. :collagen type I (red), collagen type III (green), DAPI (blue), matrix metalloproteinase 9 positive expression (bright green).. (**A**) Groups of test samples for 1W (Col. I immunofluorescence, × 40), (**B**) listed control at 1W (Col. I immunofluorescence, × 40), (**C**) test sample set 1W (Col. III immunofluorescence, × 40), (**D**) 1W of listed control (Col. III immunofluorescence, × 40), (**E**) test sample set 1W (DAPI immunofluorescence, × 40), (**F**) vehicle control at 1W (DAPI immunofluorescence, × 40), (**G**) test sample set 1W (MMP-9 immunofluorescence, × 40), (**H**) listed control 1W (MMP-9 immunofluorescence, × 40).

Immunofluorescence staining: collagen type I (red), collagen type III (green), DAPI (blue), matrix metalloproteinase 9 positive expression (bright green) were identified in Figs. 4). Matrix metalloproteinase 9 of the test sample group was mainly expressed in fibroblasts, macrophages and foreign body giant cells around the implantation site. There was no difference in the expression of matrix metalloproteinase 9 from the listed control group.

#### Subcutaneous implantation for 4 weeks

**Figure 5:**
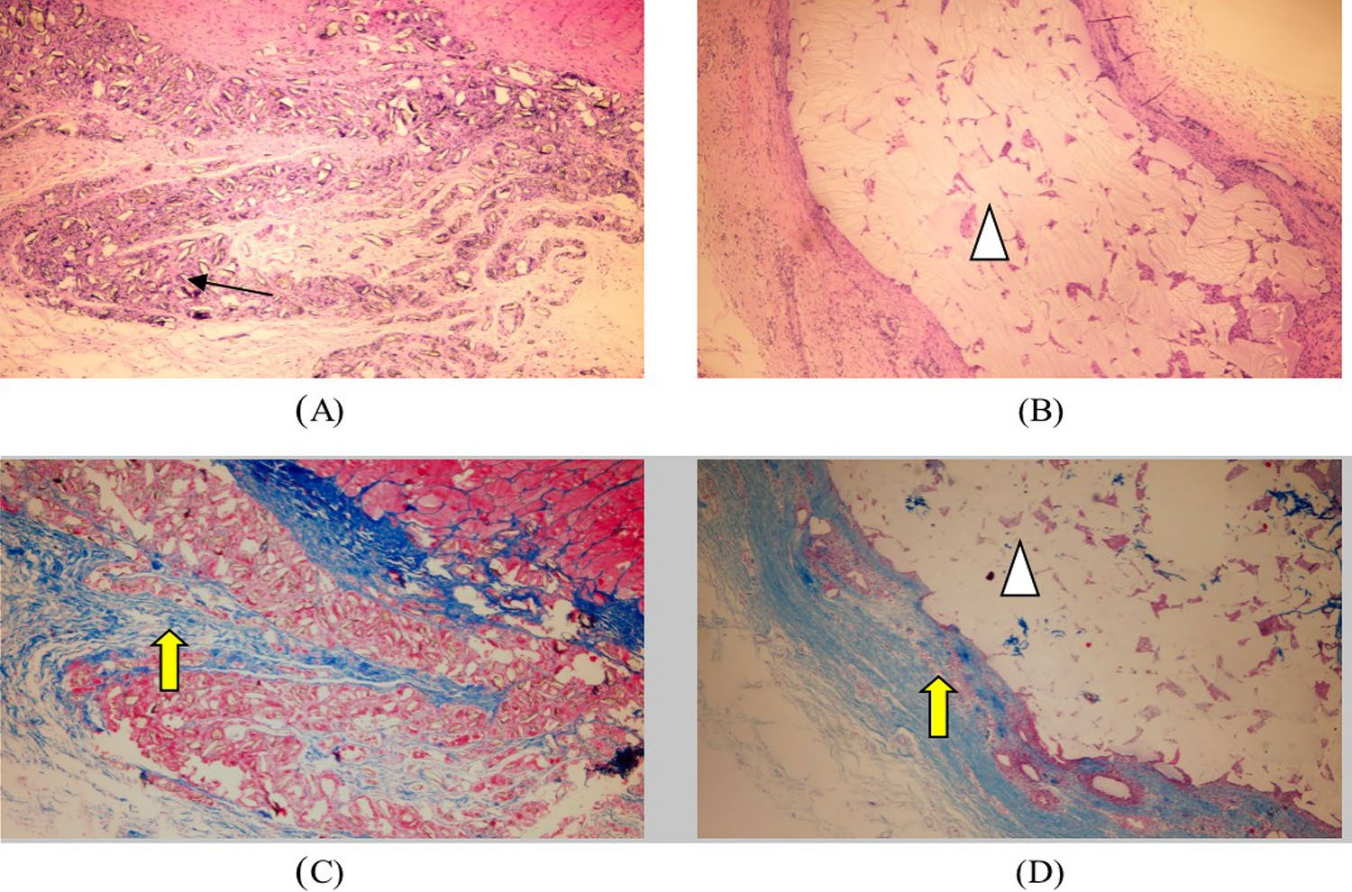
The result of Subcutaneous implantation for 4 weeks. (↑: test sample, : control sample, : collagen fibrils) . (**A**) The test sample set 4W (HE, × 40), (**B**) listed control group 4W (HE, × 40), (**C**) test sample set 4W (Masson, × 40), (**D**) listed control 4W (Masson, × 40).

Hematoxylin eosin staining: a large number of scattered particles of test samples can be seen under the microscope observation (↑ identification in Fig. 5). A small number of them are phagocytosed by macrophages, surrounded by some inflammatory cells, macrophages and lymphocytes, the volume has no noticeable change compared with before. Microscopic examination revealed a large number of gels distributed in the loose connective tissue of the subcutis (identified in Fig. 5), a few phagocytosed by macrophages and a surrounding infiltration by a few inflammatory cells, macrophages and lymphocytes, surrounded by loose fibrous tissue and no significant change in volume compared to before.

Masson trichrome staining: collagen fibrils appear blue (identified by yellow arrows in Fig. 5). The collagen fibrils in the test sample group were more numerous and more disorderly arranged at 4 weeks after surgery than at 1 week. In the marketed control group, collagen fibers were more numerous at 4 weeks after surgery than at 1 week. They were mainly distributed at the periphery of implant sites, with a small distribution between gels and more consistent alignment.

**Figure 6:**
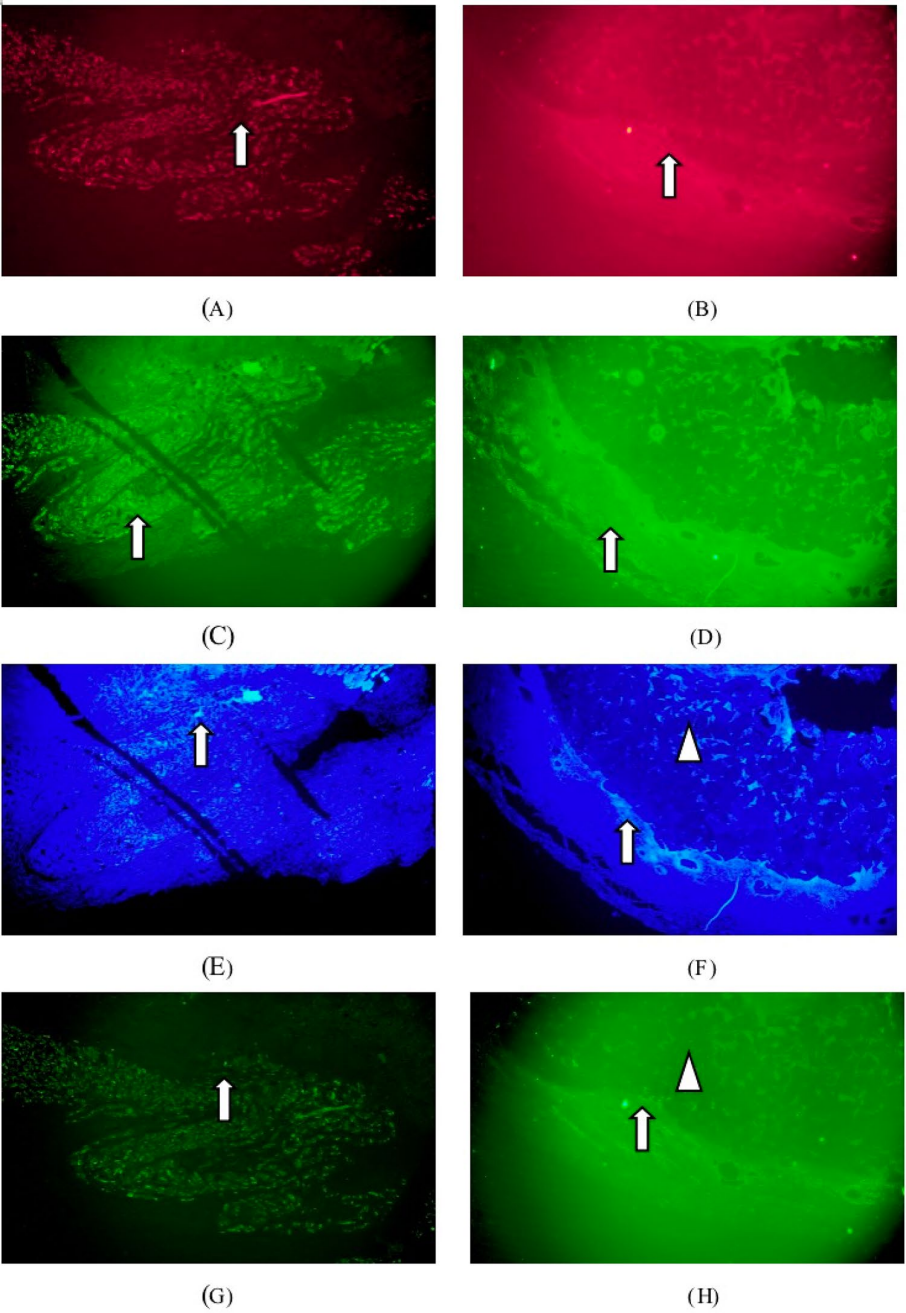
Immunofluorescence staining 4-week after implantation.  :collagen type I (red), collagen type III (green), DAPI (blue), matrix metalloproteinase 9 positive expression (bright green). (**A**) Groups of test samples for 4 W (Col. I immunofluorescence, × 40), (**B**) listed control at 4 W (Col. I immunofluorescence, × 40), (**C**) test sample set 4W (Col. III immunofluorescence, × 40), (**D**) 4W of listed control (Col. III immunofluorescence, × 40), (**E**) test sample set 4W (DAPI immunofluorescence, × 40), (**F**) vehicle control at 4W (DAPI immunofluorescence, × 40), (**G**) test sample set 4W (MMP-9 immunofluorescence, × 40), (**H**) listed control 4W (MMP-9 immunofluorescence, × 40).

Immunofluorescence staining: collagen type I (red), collagen type III (green), DAPI (blue), matrix metalloproteinase white arrows identified 9 positive expressions (bright green) in Fig. 6. Matrix metalloproteinase 9 in the test sample set was mainly expressed in fibroblasts, macrophages and foreign body giant cells around the implantation site, which did not change significantly at 1 week. Matrix metalloproteinase 9 expressions in the marketed control group were less marked than the 1-week change.

#### Subcutaneous implantation for 13 weeks

**Figure 7:**
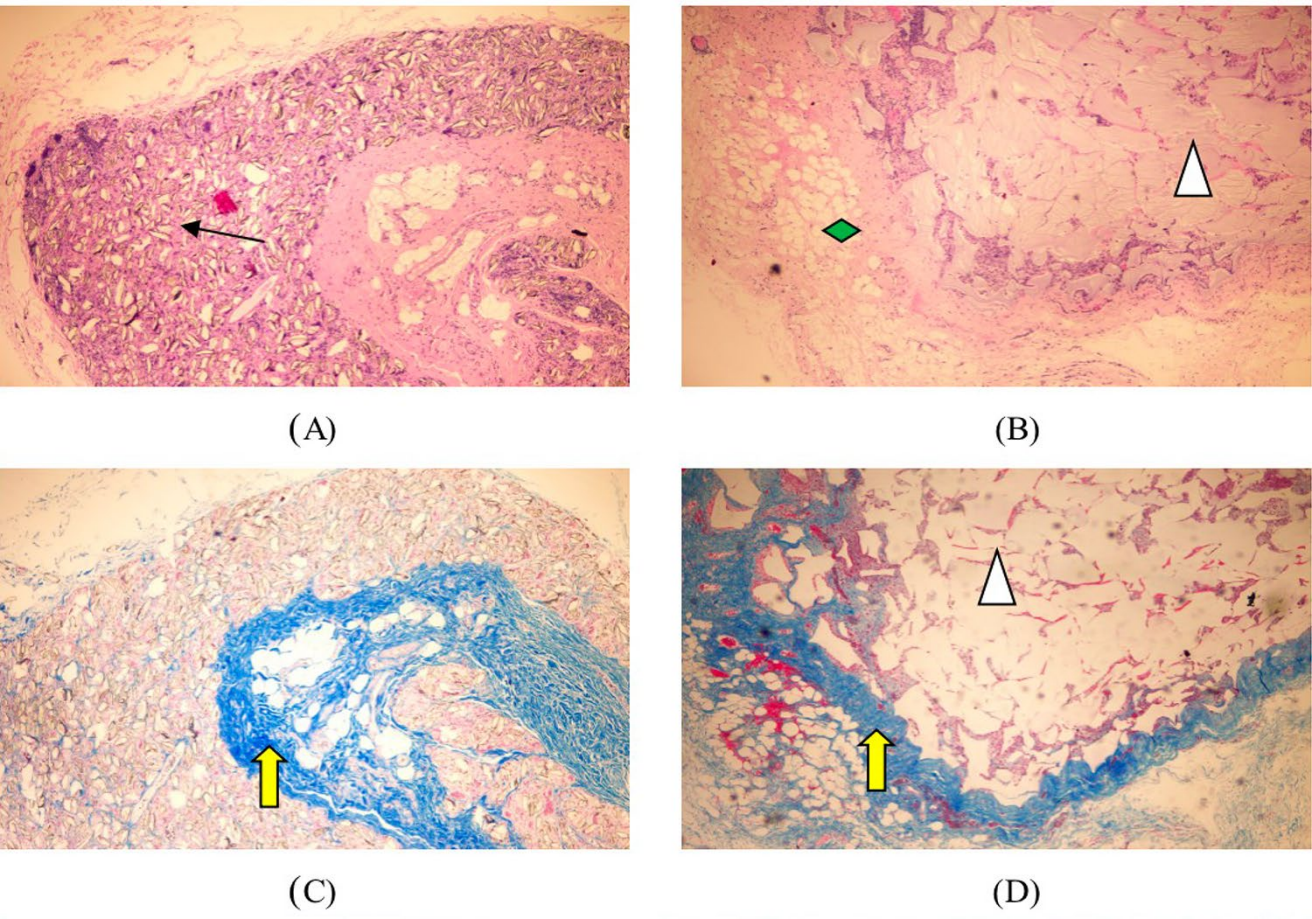
The result of Subcutaneous implantation for 13 weeks. (↑: test sample,  :control sample,  :collagen fibrils,  :adipose tissue). (**A**) The test sample set 13W (HE, × 40), (**B**) listed control group 13W (HE, × 40), (**C**) test sample set 1W (Masson, × 40), (**D**) listed control 13W (Masson, × 40).

Hematoxylin eosin staining: a large number of scattered particles of test samples are still visible under microscopic observation (↑ identification in Fig. 7), infiltrated by some lymphocytes, macrophages and foreign body giant cells, a small number of particles are phagocytosed by macrophages, during the small vessel formation (identification in Fig. 7), fibrous tissue can be seen in the periphery, and the volume changes little compared with before. Microscopically, a large number of gels distributed in the loose connective tissue of the subcutis (identified in Fig. 7), a few phagocytosed by macrophages, surrounded by some lymphocytes, macrophages, and foreign body giant cells, surrounded by loose fibrous tissue and adipose tissue (identified in Fig. 7), were observed, with no significant change in volume compared with before.

Masson trichrome staining: collagen fibrils appear blue (identified by yellow arrows in Fig. 7). The test sample group had significantly more collagen fibers in the periphery and intergranular spaces of the implant sites, which were of high density, more consistent arrangement, and deeper staining, consistent with the performance of the marketed control group. In the listed control group, collagen fibrils further increased, arranged more consistently, and stained darker.

**Figure 8:**
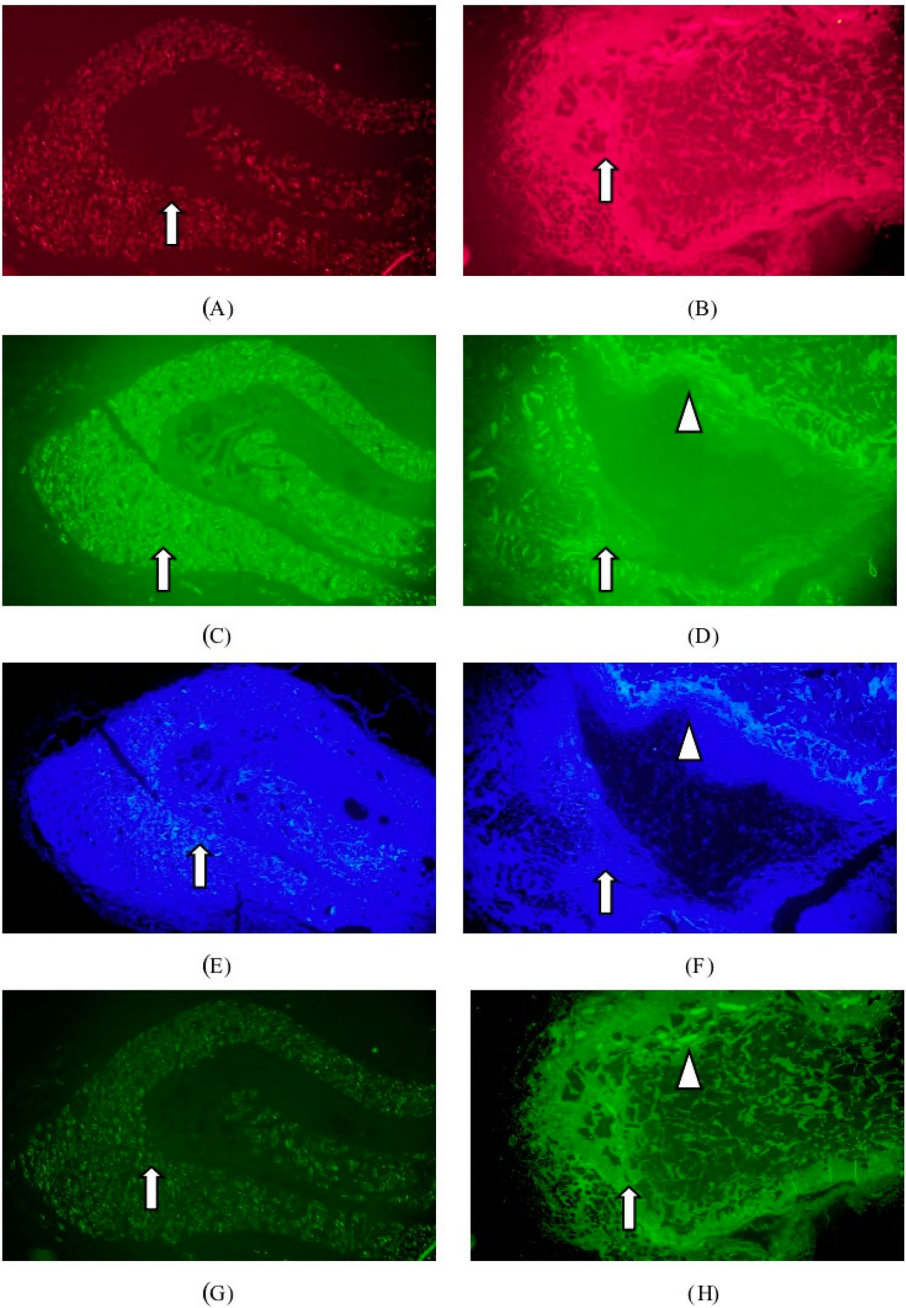
Immunofluorescence staining 13-week after implantation. :collagen type I (red), collagen type III (green), DAPI (blue), matrix metalloproteinase 9 positive expression (bright green).. (**A**) Groups of test samples for 13W (Col. I immunofluorescence, × 40), (**B**) listed control at 13W (Col. I immunofluorescence, × 40), (**C**) test sample set 13W (Col. III immunofluorescence, × 40), (**D**) 13W of listed control (Col. III immunofluorescence, × 40), (**E**) test sample set 13W (DAPI immunofluorescence, × 40), (**F**) vehicle control at 13W (DAPI immunofluorescence, × 40), (**G**) test sample set 13W (MMP-9 immunofluorescence, × 40), (**H**) listed control 13W (MMP-9 immunofluorescence, × 40).

Immunofluorescence staining: collagen type I (red), collagen type III (green), DAPI (blue), matrix metalloproteinase white arrows identified 9 positive expressions (bright green) in Fig. 8. Matrix metalloproteinase 9 positive expression was enhanced in the tested sample group, while the expression was slightly increased in macrophages, foreign body giant cells and fibroblasts surrounding the sample particles. Matrix metalloproteinase 9 (MMP-9) expression in fibroblasts and macrophages slightly increased in the marketed control group as tissue repair progressed.

#### Subcutaneous implantation for 52 weeks

**Figure 9:**
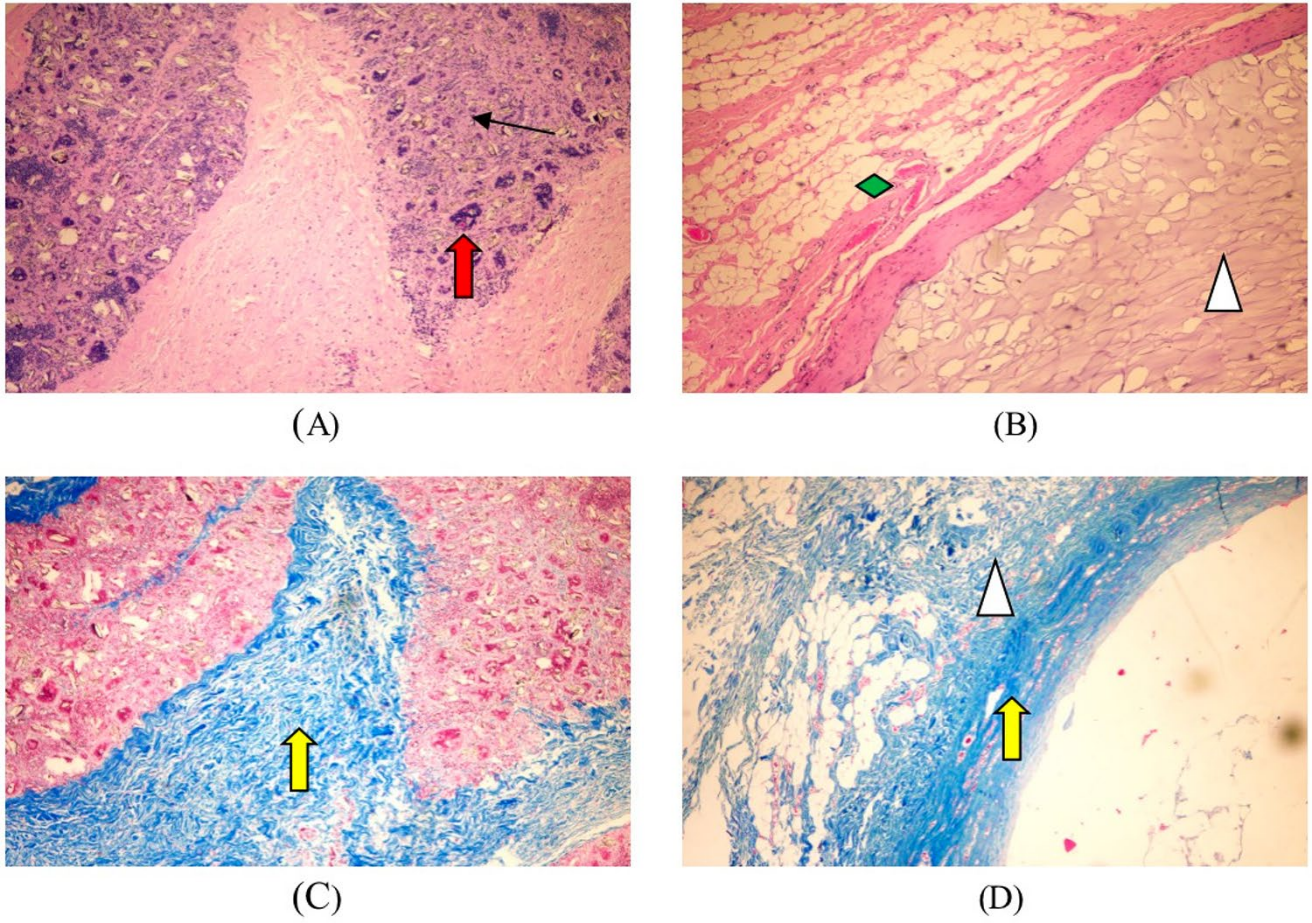
The result of Subcutaneous implantation for 52 weeks. (↑: test sample,  :control sample,  :collagen fibrils,  :adipose tissue). (**A**) The test sample set 52W (HE, × 40), (**B**) listed control group 52W (HE, × 40), (**C**) test sample set 52W (Masson, × 40), (**D**) listed control 52W (Masson, × 40).

Hematoxylin eosin staining: a large number of scattered test sample particles (↑ identification in Fig. 9) and a large number of foreign body giant cells phagocytosed with the sample particles (identification in Fig. 9) are still visible under microscopic observation, the number of sample particles is reduced compared with before, and fibrous tissue can be seen around. Microscopically, the gel is still visible (identified in Fig. 9), surrounded by dense fibrous and adipose tissue (identified in Fig. 9), and reduced in volume.

Masson trichrome staining: collagen fibers appear blue (identified by yellow arrows in Fig. 9). The experimental sample group had more collagen fibers in the periphery and intergranular area of the implantation site than before, with higher density, more consistent arrangement, and deeper staining. In the listed control group, collagen fibrils further increased, arranged more consistently, and stained darker.

**Figure 10:**
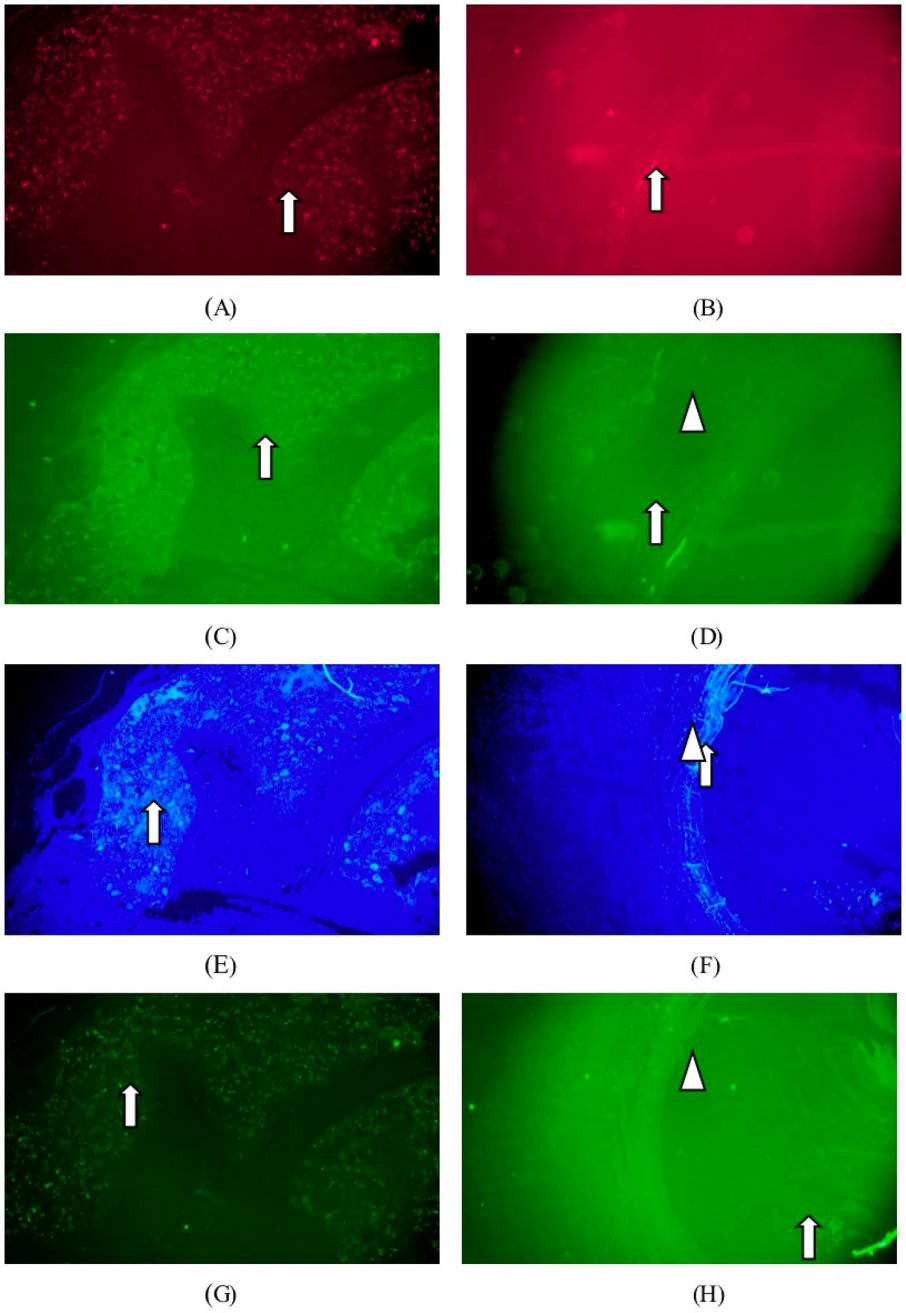
Immunofluorescence staining 52-week after implantation. :collagen type I (red), collagen type III (green), DAPI (blue), matrix metalloproteinase 9 positive expression (bright green).. (**A**) Groups of test samples for 52W (Col. I immunofluorescence, × 40), (**B**) listed control at 52W (Col. I immunofluorescence, × 40), (**C**) test sample set 52W (Col. III immunofluorescence, × 40), (**D**) 52W of listed control (Col. III immunofluorescence, × 40), (**E**) test sample set 52W (DAPI immunofluorescence, × 40), (**F**) vehicle control at 52W (DAPI immunofluorescence, × 40), (**G**) test sample set 52W (MMP-9 immunofluorescence, × 40), (**H**) listed control 52W (MMP-9 immunofluorescence, × 40).

Immunofluorescence staining: collagen type I (red), collagen type III (green), DAPI (blue), matrix metalloproteinase white arrows identified 9 positive expressions (bright green) in Fig. 10. The positive matrix metalloproteinase 9 expression in the periphery of the particles, macrophages, foreign body giant cells, and fibroblasts in the test sample group was significantly increased compared with that before. As tissue repair progressed, the expression of MMP-9 in fibroblasts and macrophages of the marketed control group changed less.

2.4 We applied Image J (V1.8.0.112) to analyze the fluorescence intensity (Mean gray value) of the experimental and control group samples.

## Discussion

Matrix metalloproteinase 9 is an essential member of the matrix metalloproteinase family and functions primarily to degrade and remodel the dynamic balance of the extracellular matrix, playing diverse roles in tumor growth, invasion and metastasis, and tissue remodeling in inflammatory diseases^7,8^. Studies show that matrix metalloproteinase-9 is highly expressed within macrophages on biomaterial surfaces and is also involved in macrophage fusion, foreign-body giant cell formation, and foreign-body response^9^. In this study, macrophages phagocytose a small portion of test sample particles can be seen after 13 weeks, a large number of foreign body giant cells engulfed with test samples can be seen after 52 weeks. Macrophages phagocytose a large number of listed control samples can be seen after 13 weeks, a small number of macrophages can be seen after 52 weeks, and the gels were surrounded by dense fibrous tissues and adipose tissues, were also further supported this notion. In this study, the positive expression of MMP 9 was mainly distributed in the cyst walls formed at the periphery of the implantation sites with fibrous encapsulation and degraded debris, and the positive cells were mainly macrophages and fibroblasts, which are the main cellular components involved in the host response and tissue repair process^10^, so the differences in MMP 9 expression could reflect the changes in the process of tissue remodeling after implantation of materials.

The collagens that make up human dermal tissue are mainly produced by fibroblast secretion, mainly type I collagen, whereas type III collagen is mainly found in infant skin. The collagen composition of adult skin is 80% type I and only 20% type III. Type III collagen is present in 80% of normal infant skin and decreases continuously with growth and development, as does type I collagen. Human facial morphology consists of a round beep-like appearance in infancy that evolves into a compact, well-delineated state of skin with the addition of type I collagen, which is the most abundant elastic collagen in humans and forms large eosinophilic fibrils called collagen fibrils, type I collagen has good mechanical properties. Its reticular architecture provides skin protection and elasticity. The skin of adults is more elastic and supportive than that of infants. The content of type I collagen in the skin decreases with age. If type I collagen synthesis in the dermis is insufficient or disrupted too much, it will weaken skin elasticity and trigger aging symptoms such as wrinkles and laxity. Therefore, protecting and supplementing type I collagen is particularly important for cosmetic skin care. In this study, the number of fibroblasts positive for matrix metalloproteinase 9 expression increased as the tissue repair progressed, so type I collagen and type III collagen gradually increased after implantation in the test samples and the marketed control samples, and type I collagen mainly existed in the interior of the fibrous capsule. In contrast, type III collagen predominated in the periphery, and the alignment gradually turned from disordered to uniform, which also explained the mechanism of increasing the elastic cosmetic outcome of skin declared by the injection of cosmetic fillers.

The difference between injectable fillers of the polylactide class and others is that, in addition to directly stimulating collagen production, it also takes some time to slowly come to mind, most appropriate for those who feel that a sudden change will be too noticeable and hope for gradual improvement and that the cosmetic filling effect can be maintained for more than two years. In this study, the 13 weeks listed foreign body reaction of control samples was more vigorous than the test samples, the 52-week one was more vigorous, and the listed foreign body reaction of control samples was smooth. This indicates that the new injectable filler has a longer degradation time and a slower appearance of a vigorous foreign body response, stimulating collagen production slightly later. With the progress of tissue repairing, the matrix metalloproteinase 9 positive expression gradually increased, and the increased positive expression of test samples in the 52-week was evident. In contrast, the change in positive expression in the marketed control samples was not significant, which also illustrated the problem of the extended maintenance time of the cosmetic filling effect.

This study is only a preliminary exploration of the tissue response and tissue remodeling mechanism of novel injectable cosmetic fillers from the fields of histology and immunology, but further research needs to be done combining other methods such as molecular, imaging, and so on to establish a quality evaluation method and research system on the tissue remodeling mechanism, to solve the bottleneck of the critical technical deficiency in the evaluation of this type of products, and to improve the overall competitiveness and level of absorbable regenerative medical device industry in China.

## Materials and methods

### Animals

24 conventional male New Zealand rabbits with weight 2.5–3.0 kg were bought from Jinan Jinfeng laboratory animal Co., Ltd with license number: SCXK(鲁)20,180,006. Animals were individually housed in stainless steel suspended cages identified by a card indicating the animal information. Conventional environment with license number: SYXK (鲁) 20,190,013, applicable to conventional rabbit. The temperature range was 16 ~ 26 °C. The humidity range was 40–70%. The light cycle was controlled (12 h light, 12 h dark). Rabbits were feed with Rabbit Maintenance Feed bought from Beijing KeaoXieli Feed Co., Ltd with license number: SCXK(京)2019–0003. The protocol and any amendments or procedures involving the care or use of animals on this study were reviewed and approved by Institutional Animal Care and Use Committee Laboratory Animals (IACUC) of the test facility. Husbandry conditions conformed to “Management Procedures of Animal Laboratory and Laboratory Animals” and “Standard Operating Procedures of Rabbit Husbandry” of the test facility. Experiment conformed to “Standard Operating Procedures of Tests for Local Effects after Implantation” of the test facility. The test facility has agreed us to participate in this study and submit the manuscripts.

### Test article

The test article was from an unlisted product provided by a company. The main component is poly (L-lactic acid), which the sponsor supplemented. The listed control sample is boric acid from a company's marketed product, whose main component is cross-linked sodium hyaluronate. Negative control sample: HDPE from Hatano Research Institute Food and Drug Safety Center^11^.

### Experimental procedure

Each rabbit was clipped free of the fur from the back and both sides of the spinal column to yield a sufficient implant area before the treatment. The animals were anesthetized by intravenous of pentobarbital sodium dosed. The surgical site was wiped with tincture of iodine. Test articles were implanted into subcutaneous tissue along one side of the spine 2.5 cm from the midline and parallel to the spinal column, and four test articles were implanted into one side with about 2.5 cm apart from each other. Negative control sampleswere implanted into the other side of the subcutaneous tissue of 12 rabbits. In the same way, the marketing control samples and negative control samples were implanted into the subcutaneous tissues of another12 animals. One week, four weeks, thirteen weeks and fifty-two weeks after implantation the rabbits were euthanized, the implant articles and the enough well tissue around it were excised and preserved in 10% phosphate neutral buffered formalin at least 48 h. After fixation, and Hematoxylin–Eosin staining, Masson trichrome staining and immunofluorescence staining were performed to characterize the local effects in vivo and the expression of type I collagen (Col. I), type III collagen (Col.III) and matrix metalloproteinase 9 (MMP-9). The inflammatory reaction, the formation of collagen, the degree of fibrosis and the degradation of materials were observed and evaluated under the microscope^12^.

### Statistical method

Apply SPSS 13.0 software for statistical analysis of experimental data. The experimental results are presented in ${\overline{\text{x}}} \pm {\text{s}}$.

### Ethics approval and consent to participate

The protocol and any amendments or procedures involving the care or use of animals on this study were reviewed and approved by Institutional Animal Care and Use Committee Laboratory Animals (IACUC) of Shandong Institute of Medical Device and Pharmaceutical Packaging Inspection. Husbandry conditions conformed to “Management Procedures of Animal Laboratory and Laboratory Animals” and “Standard Operating Procedures of Rabbit Husbandry” of the test facility. Experiment conformed to “Standard Operating Procedures of Tests for Local Effects after Implantation” of the test facility. The test facility has agreed us to participate in this study and submit the manuscripts.

## Data Availability

The raw data/protocol/final report have been preserved in the archives of National Medical Products Administration Jinan Quality Supervision and Inspection Center for Medical Devices. If someone wants to request the data from this study Dr. Sunlikui should be contacted. (Email:sunlikui2002@126.com).
